# Emerging Screening Approaches in the Development of Nrf2–Keap1 Protein–Protein Interaction Inhibitors

**DOI:** 10.3390/ijms20184445

**Published:** 2019-09-10

**Authors:** Chung-Hang Leung, Jia-Tong Zhang, Guan-Jun Yang, Hao Liu, Quan-Bin Han, Dik-Lung Ma

**Affiliations:** 1 State Key Laboratory of Quality Research in Chinese Medicine, Institute of Chinese Medical Sciences, University of Macau, Macao 999078, China (J.-T.Z.) (G.-J.Y.); 2 Department of Chemistry, Hong Kong Baptist University, Kowloon Tong, Hong Kong 999077, China; 3 College of International Education, School of Continuing Education, Hong Kong Baptist University, Shek Mun, Hong Kong 999077, China

**Keywords:** Keap1–Nrf2, protein–protein interaction, inhibitors, oxidative stress, virtual screening

## Abstract

Due to role of the Keap1–Nrf2 protein–protein interaction (PPI) in protecting cells from oxidative stress, the development of small molecule inhibitors that inhibit this interaction has arisen as a viable approach to combat maladies caused by oxidative stress, such as cancers, neurodegenerative disease and diabetes. To obtain specific and genuine Keap1–Nrf2 inhibitors, many efforts have been made towards developing new screening approaches. However, there is no inhibitor for this target entering the clinic for the treatment of human diseases. New strategies to identify novel bioactive compounds from large molecular databases and accelerate the developmental process of the clinical application of Keap1–Nrf2 protein–protein interaction inhibitors are greatly needed. In this review, we have summarized virtual screening and other methods for discovering new lead compounds against the Keap1–Nrf2 protein–protein interaction. We also discuss the advantages and limitations of different strategies, and the potential of this PPI as a drug target in disease therapy.

## 1. Introduction

**Figure 1:**
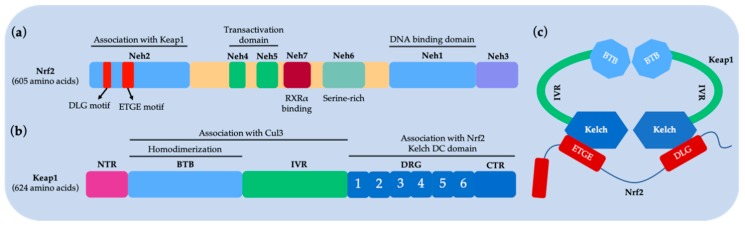
Schematic diagram of Nrf2 and Keap1 domains. (**a**) Nrf2 comprises seven major domains, called Neh1–Neh7. Neh1, the basic region of the leucine zipper motif, regulates the stability, DNA-binding, and Maf dimerization. Neh2 possesses two binding regions, DLG motif (DLG) and ETGE tetrapeptide motif (ETGE), that are responsible for interacting with Keap1. Neh4, Neh5 and Neh3 regulate the transactivation of Nrf2. The serine-rich Neh6 regulates Nrf2 stability; (**b**) Keap1 comprises three major domains. BTB regulates Keap1 homodimerization and binding with Cul3. IVR possesses a key cysteine residue that links the BTB domain to the C-terminal Kelch/DGR domain. The Kelch/DGR domain regulates binding of Nrf2 to Neh2; (**c**) The two-site binding model of the Keap1–Nrf2 PPI (referred to as hinge and latching models). Under steady-state conditions, the Keap1 homodimer uses its two Kelch domains to bind to a single Nrf2 molecule via its DLG and ETGE domains.

Oxidative stress is associated with the pathogenesis of cancers, neurodegenerative diseases, inflammatory diseases, cardiovascular diseases and aging, which are major causes of human death [1]. Oxidative stress occurs due to the production of reactive oxygen species (ROS) such as superoxide and hydrogen peroxide, as well as reactive nitrogen species (RNS), causing damage to cells and inflammation [2,3,4]. Cells possess antioxidant defense systems that help protect against oxidative damage [5,6]. These defenses include many phase II enzymes or reductants such as quinone oxidoreductase 1 (NQO-1), NADPH, glutathione, glutamate–cysteine ligase (GCL), superoxide dismutase (SOD), catalase, glycopeptide peroxidase (GPx), thioredoxin (TRX), heme oxygenase-1 (HO-1) and glutathione S-transferase (GST) [7,8]. Typically, these defenses are activated by the binding of the upstream antioxidant response element (ARE) by transcription factors such as nuclear factor erythrocyte 2 related factor 2 (Nrf2) [9,10]. Nrf2 is a cap’n’collar (CNC) basic region leucine zipper (bZIP) transcription factor that controls over 100 oxidative stress-associated genes by recognizing the ARE enhancer sequence (5’-GTGACnnnGC-3’) (Figure 1) [11].

**Figure 2:**
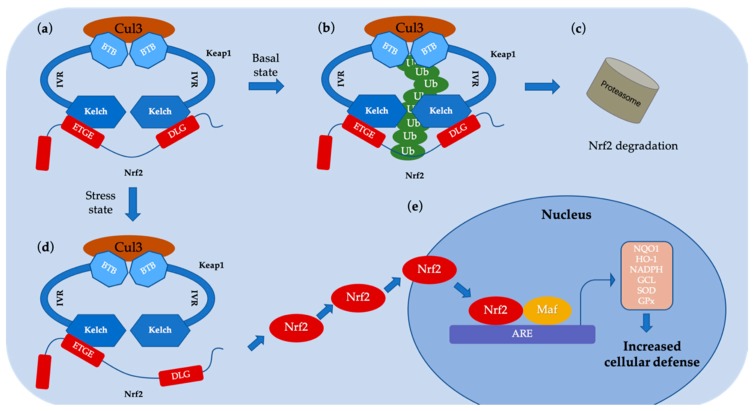
Schematic representation of the Nrf2–Keap1 signaling mechanism. (**a**, **b** and **c**) Under normal conditions, Keap1 interacts with the ETGE and DLG motifs on Nrf2 and allows Nrf2 to enter the Keap1–Cul3 ubiquitin ligase complex, resulting in the ubiquitination and then degradation of Nrf2; (**d**) Under stress conditions, the inducer modifies the key cysteine residue of Keap1, leading to the repression of Nrf2 ubiquitination by dissociation inhibitory complexes. In the hinge and latch model, modification of as specific Keap1 cysteine residue results in a conformational change in Keap1, leading to the release of the Nrf2 DLG motif from Keap1. Nrf2 ubiquitination is prevented, but binding to the ETGE domain is still retained, resulting in Nrf2 escaping from the ubiquitination system; (**e**) Nrf2 protein bypasses Keap1 and moves into the nucleus, interacts with the ARE and stimulates Nrf2 targets. Transcription of genes including quinone oxidoreductase 1 (NQO1), heme oxygenase-1 (HO-1), nicotinamide adenine dinucleotide phosphate (NADPH), glutamate–cysteine ligase (GCL), superoxide dismutase (SOD), glycopeptide peroxidase (GPx), increases the defense of cells against oxidative stress.

Kelch-like ECH-associated protein 1 (Keap1) is a protein rich in cysteine that acts as a major regulator of Nrf2 and mediates oxidative stress [11,12,13]. Under normal stress-free conditions, Keap1 is an adapter for the Cullin3 (Cul3)-based ubiquitin E3 ligase complex [14]. In this ternary complex, Keap1 not only bridges Cul3 and Nrf2 together via protein–protein interactions (PPI), but also functions as a switch for Nrf2 ubiquitination machines (Figure 2) [15]. This complex targets Nrf2 to the proteasome for degradation, which limits the basal levels of Nrf2 activity [16]. Under oxidative stress conditions, Keap1 is inactivated, causing Nrf2 to be released from Keap1. Free Nrf2 subsequently moves to the nucleus and activates ARE-dependent antioxidant groups to protect cells from oxidative damage [17]. Thus, the Keap1–Nrf2–ARE signaling cascade is a potential pharmacological target for oxidative disorders.

The 605-residue human Nrf2 contains seven highly conserved domains (Neh1 to Neh7) with different functions [18,19] (Figure 1a). Neh1 is a basic leucine zipper motif that allows for heterodimer formation to DNA, via partnering with small muscle decidual fibrosarcoma (Maf) protein or another transcriptional partner [20]. Neh2 holds two motifs, DLG and ETGE, that are critical for the interaction between Keap1 and Nrf2 for regulating the ubiquitination and stability of Nrf2 [21]. Neh3 is responsible for the transactivation of ARE-dependent transcripts [22]. Neh4 and Neh5 interact with the CREB-binding protein (CBP), a transcriptional coactivator that controls the transcription activity of Nrf2 [23]. The serine-rich Neh6 motif affects the stability of Nrf2 in a Keap1-independent fashion [24]. Neh7 binds to retinoic acid X receptor alpha (RXRα) to inhibit the Nrf2–ARE signaling system [25]. The seven cysteines of Keap1 (C151, C257, C273, C288, C297, C434 and C613) are involved in the redox sensing and activation of Nrf2. Human Keap1 has five domains: the N-terminal region (NTR), the broad-complex, tramtrack and bric a brac (BTB) domain, an intervening region (IVR) with a double glycine region (DGR), multiple cysteines, and a C-terminal region (CTR) [26]. The BTB domain can be dimerized with Cullin3 (Cul3), which regulates Nrf2 ubiquitination [27]. The IVR domain has a reactive cysteine that acts as a sensor for oxidative stress [28]. The DGR domain contains six repeating Kelch motifs. The DGR and CTR domains are together called the DC domains and bind to Neh2 of Nrf2 to regulate the interaction between Keap1 and Nrf2.

Nrf2 activators have been developed as potential therapeutic molecules to combat oxidative stress [29]. These activators are metabolically activated to become electrophiles which target the cysteine residues of Keap1, resulting in the separation of the Nrf2–Keap1 complex [30]. However, since the mechanism of action of these compounds involves covalent targeting of cysteine thiols, they are not selective for Keap1 over other cysteines prevalent in cells [31]. Thus, Nrf2 activators may perturb multiple cellular targets, leading to unpredictable side effects. An alternative method of activating Nrf2 is through the non-covalent targeting of the Keap1–Nrf2 PPI, which is a strategy that potentially offers higher safety and efficacy.

## 2. Diseases Related to the Nrf2–Keap1 Protein–Protein Interaction

To develop inhibitors of the Nrf2–Keap1 PPI, we must first understand the molecular mechanism by which Keap1 interacts with Nrf2. The Neh2 domain of Nrf2 holds two binding regions, ETGE and DLG, which interact with the C-terminal domain(DC)region of Keap1 with distinct strengths [32]. These two motifs are included into the polylysine region, which is indispensable for ubiquitination in the so-called “hinge and latch” mechanism [33]. In this model, the higher-affinity ETGE region acts as the “hinge” to immobilize Nrf2 to Keap1, while the lower-affinity DLG region acts as a “lock” to promote Cul3-based E3 ligase complex formation and subsequent ubiquitination [34]. Thus, inhibitors that can compete for one or both of the Nrf2 binding sites can block the Keap1–Nrf2 interaction, thereby enhancing Nrf2 activity.

### 2.1. Cancer

As a transcription factor, Nrf2 is involved in the control of phase II genes that express enzymes responsible for detoxifying chemical carcinogens [35]. Nrf2 activity can prevent oxidative stress and aid in chemotherapy or radiation therapy [36,37,38]. Studies in Nrf2 knockout mice (Nrf2^−/−^) indicate that Nrf2 prevents the formation of tumors in the stomach, intestines and skin by chemical carcinogens. For example, the likelihood of gastric tumors in Nrf2-deficient mice following exposure to the carcinogen benzo(a)styrene is greatly increased compared to wild-type mice [39]. Meanwhile, intestinal tumors were increased in Nrf2-deficient mice challenged with nitroxethane and dextran sulfate versus wild-type animals [40]. In addition, the incidence of skin tumors in Nrf2-deficient mice was significantly increased after exposure to the potent carcinogen 7,12-dimethylaniline compared to wild-type mice [41]. Therefore, small molecule antagonists of the Nrf2–Keap1 PPI hold great potential in combating oxidative stress for treating cancer.

### 2.2. Neurodegenerative Disease

The central nervous system is highly susceptible to oxidative stress. ROS produced during oxidative stress damages lipids, proteins and DNA, leading to the formation of abnormal protein aggregates, synaptic nuclear protein variants, mitochondrial dysfunction and microglia activation. Hence, oxidative stress is a key contributor to neurodegenerative conditions, such as Huntington’s disease (HD) and Parkinson’s disease (PD). The main symptoms of PD are mostly due to the degradation of dopaminergic neurons in the substantia nigra pars compacta, and the accumulation of alpha-synuclein in the cytoplasm of neurons in various regions of the brain in the form of Lewy bodies [42]. Activation of the Nrf2–ARE system would lead to increase of NQO-1 and HO-1 by nigral immunoreactivity [43]. The protective effect of Nrf2 is demonstrated by the observation that the highly neurotoxic dopamine analogue, 6-hydroxydopamine (6-HAD), activates Nrf2 to upregulate ARE and activates cellular defense mechanisms preventing oxidative stress [44]. Meanwhile, Huntington’s disease (HD) is mainly characterized by decreased cognitive ability in the form of chorea movement and behavioral difficulties [45]. In the primary stage of HD, the activation of Nrf2 promotes the overexpression of important cytoprotective genes in the Keap1–Nrf2–ARE pathway to activate astrocytes and microglia, thereby protecting the brain from ROS damage [46]. Therefore, activation and overexpression of Nrf2 by targeting the Nrf2–Keap1 PPI is a promising pharmacological target for treating neurodegenerative diseases.

### 2.3. Diabetes

Diabetes and diabetic complications are metabolic disorders caused by hyperglycemia-induced oxidative stress. Nrf2 acts as a defense against diabetic complications via decreasing hyperglycemia-mediated oxidative and nitrosative stress and mitigating kidney damage [47]. Moreover, pre-diabetic and diabetic patients showed diminished Nrf2 expression [48]. It has also been suggested that Nrf2 has a protective function in the diabetic complications of nephropathy, and can be targeted for prevention or progression of the disease [49]. The protective function of Nrf2 in regulating metabolism and blood sugar concentration has raised attention in targeting this protein for the treatment of diabetes and its complications.

### 2.4. Other Diseases

Apart from the above mentioned diseases, the protective effects of Nrf2 have been studied in other conditions. Nrf2 can play a role in cardiovascular disease (CVD) and its complications, including atherosclerosis, hypertension and cardiomyopathy [50,51,52]. Nrf2 has ubiquitous expression in the cardiovascular system and has a key role in regulating cardiovascular homeostasis by inducing ARE [53]. Meanwhile, loss or consumption of Nrf2 expression exacerbates lung toxicity due to various sources of oxidation, leading to respiratory diseases [54]. In addition, Nrf2 is associated with the pathogenesis of liver disease and hepatotoxicity, and is considered to be the key to combating liver damage [55]. All these studies indicate that the Keap1–Nrf2 PPI is a promising target for drug development.

## 3. Strategies in the Screening of Nrf2–Keap1 PPI Inhibitors

As the basis for screening Nrf2–Keap1 PPI inhibitors, the co-crystal structure of the Keap1 Kelch domain with Nrf2 was recently presented [26,56,57,58,59], which facilitates many emerging screening strategies for this target. Here, high-throughput screening (HTS), structure-based virtual screening (SBVS), the fragment-based approach, and other methods will be introduced (Table 1).

### 3.1. Screening Nrf2–Keap1 PPI Inhibitors Based on HTS and VS

**Figure 3:**
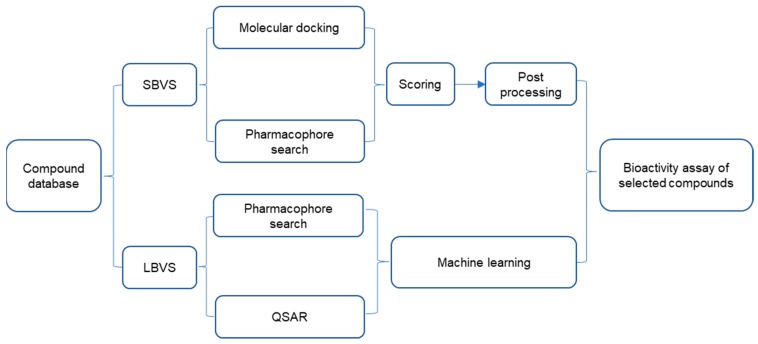
General flow chart of virtual screening. SBVS = structure-based virtual screening; LBVS = ligand-based virtual screening; QSAR = quantitative structure-activity relationship.

HTS and VS are widely used in the discovery process of lead compounds. HTS refers to the process of screening large numbers of compounds experimentally to find active hits. Meanwhile, VS uses computer technology and software to predict experimental activity in silico. After shortlisting hits, both methods will perform biological assays on the potential compounds to verify their activity. The biggest differences between the two methods lie in that HTS screens a large number of compounds based on biological evaluation (typically a few hundreds of thousands), while VS screens from millions of compounds, but the number tested biologically may be much lower (typically 10–100). This suggests that HTS may miss some inhibitors that only VS can identify, and vice versa. VS methods fall into two main categories: structure-based virtual screening (SBVS) and ligand-based virtual screening (LBVS) techniques (Figure 3). The purview of SBVS includes molecular docking and pharmacophore searching, while LBVS includes pharmacophore searching and establishing quantitative structure-activity relationship (QSAR) [29].

A number of Keap1–Nrf2 PPI inhibitors have been discovered by HTS [66]. However, VS methods have recently emerged that could greatly accelerate the discovery of new lead compounds against Keap1–Nrf2 PPI [67]. As the name implies, VS is a HTS process that is performed on a computer, and is also known as *in silico* screening [68]. VS exploits the massive calculating power available to modern components in order to screen real or virtual large compound databases against a particular target [69]. VS can also be used to produce a three-dimensional structure or QSAR model of a specific target biological macromolecule [70]. Compounds from a database can then be computationally screened for binding to the target biomacromolecules or that conform to the QSAR model, and the top hits can then be shortlisted for experimental screening studies [71]. By weeding out millions to billions of potential compounds *in silico*, the number of compounds to be screened experimentally can be greatly reduced, decreasing the time and cost required for research [72,73].

#### 3.1.1. High-Throughput Screening of Nrf2–Keap1 Protein–Protein Interaction Inhibitors

**Figure 4:**
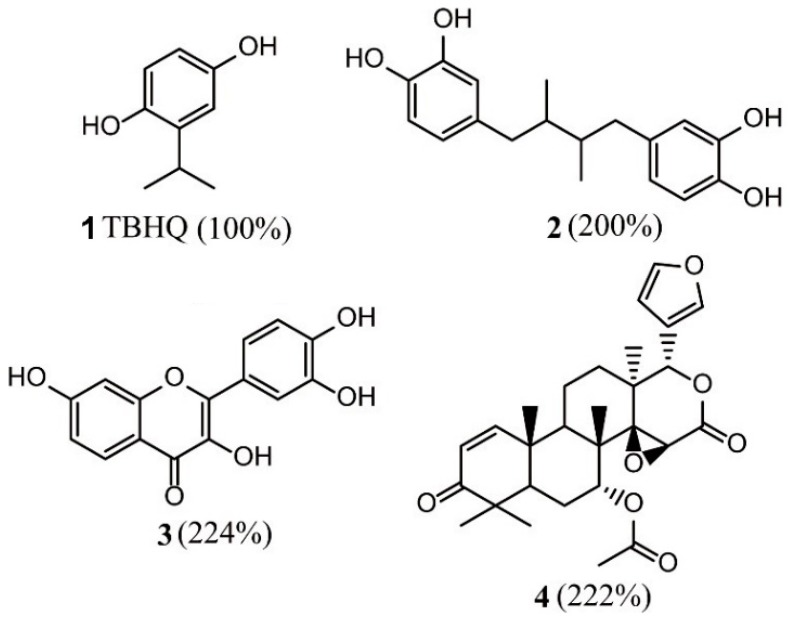
Structures of compounds **1–4**. TBHQ = tert-butylhydroquinone.

Smirnova et al. screened Nrf2 activators from a library of over 2000 compounds based on HTS and real-time monitoring of Nrf2 activators [60]. The cell-based Neh2–luciferase assay monitors the interaction of the Neh2 domain of Nrf2 with firefly luciferase (Neh2–luciferase) to detect small molecules that have the effect of activating Nrf2. The three most robust and non-toxic Nrf2 activators (compounds **2–4**) (Figure 4) were identified and all of them showed over 200% activity compared to the positive control canonical activator of Nrf2, compound **1** (tert-butylhydroquinone, TBHQ).

**Figure 5:**
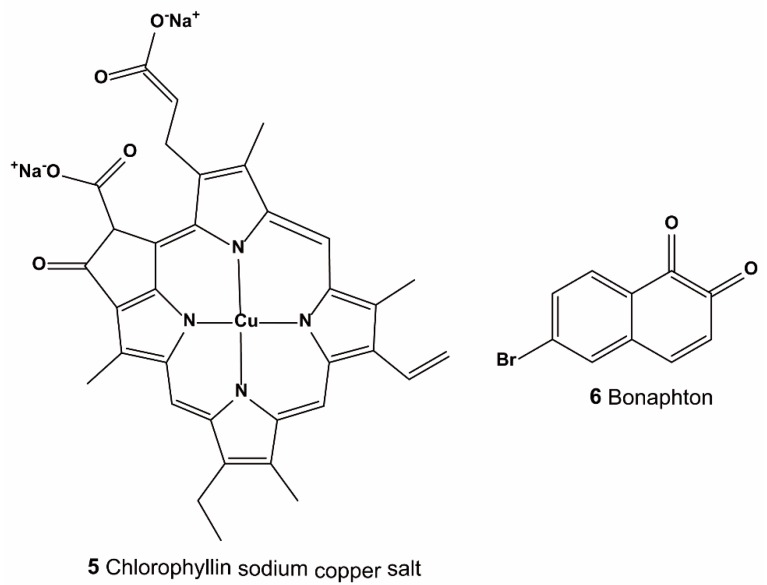
Structures of compounds **5–6**.

Yoshizaki et al. performed drug-repositioning HTS from 1633 drugs to screen Keap1–NRF2 PPI inhibitors using a fluorescence correlation spectroscopy (FCS) screening system, a ARE gene promoter assay, and a RT-qPCR assay [61]. After initial screening, 12 candidate compounds were identified. Further analysis showed that two of them (Figure 5: compound **5** and **6**) could significantly increase Nrf2 protein levels, ARE gene promoter activity, and HO-1 mRNA in HepG2 cells. The half maximal inhibitory concentration (IC_50_) values by FCS assay of compounds **5** and **6** were 35.7 μM and 37.9 μM, respectively.

#### 3.1.2. Virtual Screening

SBVS is a technique for utilizing structural information relative to a target receptor based on experimentally determined or homologous constructed three-dimensional structures of receptor biomacromolecules [74]. Molecular docking is a type of structure-based approach that attempts to predict the potential binding pose of small molecules into the target receptor. Then, the binding modes are scored by using a scoring function to calculate the binding strength of the ligand–receptor complex [75]. The basic workflow of a docking-based screening is depicted in Figure 3, and consists of the following steps: (1) establishing a receptor model: attribution and structural optimization of biological macromolecules to determine the binding sites of small molecules and constructing computational grids; (2) building a compound library: convert 2D structures into 3D structures and optimize structures to form 3D small molecule databases; (3) virtual screening: each compound in the 3D small molecule database is docked and scored in the active pocket of the biomacromolecule to assess its binding activity to the receptor; (4) processing of hit molecules: based on the scoring results, higher-ranking compounds are selected for evaluation in bioassay testing.

**Figure 6:**
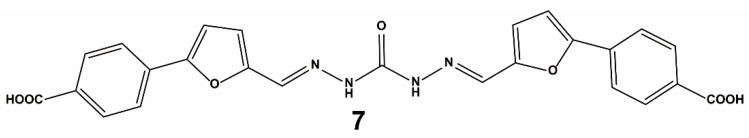
Structure of compound **7**.

Sun et al. reported the SBVS of the Specs compound database using the receptor–ligand binding model of Nrf2–Keap1 [10]. To recognize Keap1, Nrf2–Keap1 PPI inhibitors should possess a negative ionization center [56]. Therefore, the screening was restricted to compounds possessing a formal charge lower than or equal to -1 at pH 7.4. This reduced the library size from the original Specs database of 251,774 compounds to 21,199 compounds. Next, the Receptor–Ligand Pharmacophore Generation Protocol in Discovery Studio 3.0 was used to examine the important interactions between Keap1 and its binding partners. A pharmacophore was produced from the two crystal structures of the Nrf2 ETGE motif and the Kelch motif of Keap1 (Protein Data Bank (PDB) codes: 1X2R and 2FLU) by retaining superimposed and the overlapping pharmacophore features. The final pharmacophore model contained two hydrogen bond acceptors (HBA), one hydrogen bond donor (HBD), and three negative ionizable centers. Using the ligand–pharmacophore model, 2325 compounds were shortlisted. Then, the Ligandfit docking procedure was performed against the Keap1 Kelch domain (PDB ID: 4IQK) and consensus scores from seven scoring functions including DockScore, LigScore1, LigScore2, -PLP1, -PLP2, Dockscore, and -PMF were calculated to filter the compounds. The top 40% of molecules were used in the docking procedure and the 225 molecules with consensus scores equal or greater than four were further examined using the Implicit Solvent Model in PBSA software to calculate binding energies. The top 10% of molecules were selected for visual inspection for their docked pose, particularly for the presence of an electrostatic interaction between the small molecule and key arginine residues (Arg380, Arg415 and Arg483), giving a final list of 17 compounds that were selected for experimental screening against the Keap1–Nrf2 interaction. From these results, compound **7** (Figure 6) was discovered as a direct PPI inhibitor of Nrf2–Keap1 with a half maximal effective concentration (EC_50_) value of 9.80 μM in a fluorescence polarization (FP) experiment. Compound **7** also activated Nrf2 transcription in a cellular ARE–luciferase reporter assay in a dosage-dependent fashion. Docking studies showed that hydrophobic interactions promote the binding of compound **7** to Keap1. Importantly, this compound **7** activates Nrf2 with sustained low toxicity by directly blocking the interaction between Keap1 and Nrf2, versus conventional Nrf2 activators.

**Figure 7:**
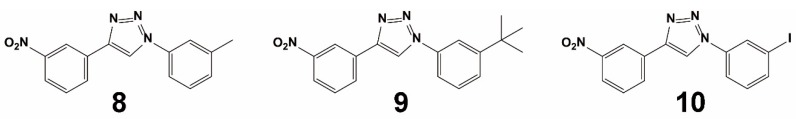
Structures of compounds **8–10**.

Bertrand et al. used the ZINC database ‘clean fragments’ sub-library (~178,000 compounds) to perform a fragment-based virtual screening campaign [62]. These molecules were subjected to docking against the C-terminal Kelch region of human Keap1 (PDB ID: 2FLU) at the “ETGE” domain of the Nrf2 peptide site. The binding site was determined, and then the molecules were ranked with Autodock 4.2 and DOCK 6.6 algorithms. The 364 hits with Autodock binding energies more negative than −8.0 kcal/mol for Keap1 were chosen for visual inspection. Interestingly, most of the hits could be classed within a small subset of molecular architectures. Inspection of the top-scoring binding poses indicated that nitro or carboxylate motifs could form desirable electrostatic or hydrogen bonding links with R380, R415, R483 and N382 of Keap1. For some compounds, additional hydrogen bonds were formed with the S602 side chain inside the binding region. The researchers finally developed a series of inhibitors that inhibit the activity of the Keap1–Nrf2 PPI based on the 1,4-diaryl-1,2,3-triazole architecture. Three compounds, **8–10** (Figure 7), could stabilize Nrf2 and induce NQO1 and HO-1 expression in a dose- and time-dependent fashion in cells, which was correlated with their potency against the Keap1–Nrf2 PPI in the FP assay. Compound **9** reversibly binds to Keap1 and is not toxic over a broad dosage range (0.1–200 μM) by directly binding to Keap1 via modified its cysteine residue (C151). In live cell imaging experiments, compound **9** drove the formation of an “open” state of the Keap1–Nrf2 complex. These new inhibitors have great potential to disrupt the protein–protein interactions of Keap1 and Nrf2.

#### 3.1.3. Combined Screening Using VS and HTS

**Figure 8:**
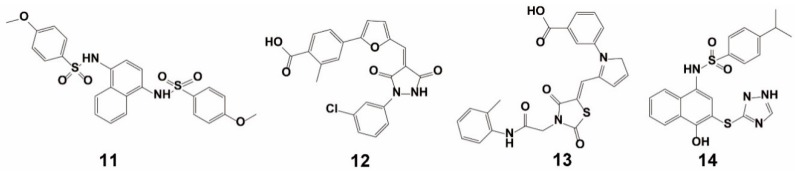
Structures of compounds **11–14.**

Zhuang et al. performed a structure-based VS and hit-based substructure search campaign using the Schrodinger’s Glide application from the Specs database of more than 300,000 molecules [64]. The database, including only organic compounds, was downloaded and prepared using Pipeline Pilot 7.0 (http://accelrys.com/products/pipeline-pilot/). For each compound, a set of physical properties was evaluated to assess its drug-likeness profile and only compounds with Lipinski’s properties were used [64], which reduced the prepared database size to 153,611 unique compounds. The grid used for docking was based on the **11**-Keap1 complex (PDB entry: 4IQK), whose ligand has the highest binding inhibition. Due to the size of the screening library of over one hundred thousand structures, the virtual screening was carried out in three successive steps, with gradually more demanding computational sampling and precision levels. The Glidescore was used to rank and to assist the selection of ligands throughout the screening process. First, high–throughput virtual screening (HTVS) with default parameters was used to eliminate the most unlikely candidates from the screening library. Only the top 10,000 ranked structures were selected for the subsequent docking screening with Standard Precision (SP). A Glidescore of –6.9 obtained for compound **11** was used as a cutoff value for the selection of the initial set of 853 compounds. The interactions of these 853 compounds with Keap1 were then individually visually inspected. The candidate compounds were chosen based on a number of filters: (1) the ligand can be inserted into the binding pocket of Keap1 with a reasonable pose; (2) there should be at least one hydrogen bond or π–π stacking interaction with Keap1; (3) considering that the Keap1 structure has a six-bladed fold symmetry [64], ligands symmetrically binding with the protein are favored. Consequently, only 376 candidate compounds that met the above criteria were selected and re-docked using the default Extra Precision (XP) protocol. Then, using the Glidescore of –6.7 obtained for compound **11** as a reference, 113 compounds with an XP Glidescore less than –6.5 were identified, among which 90 compounds were finally selected upon removing duplicates with different conformations and ionization states. The comparison used the calculated Tanimoto coefficient of 2D fingerprints, a binary expression of a set of fragment descriptors that a molecule possesses, obtained for each molecule. Biological validation resulted in the successful identification of four novel inhibitors (**11**, **12**, **13**, and **14**) (Figure 8) of the Keap1–Nrf2 PPI. These compounds showed moderate potency with equilibrium dissociation constant *(K*d) values between 2.9 µM and 15.2 μM. Moreover, the compounds showed three-fold higher cellular activity at activating Nrf2 compared to the most potent non-covalent Keap1 inhibitors known to date. Further cell-based assays confirmed that these three inhibitors disrupted the PPI of Keap1 and Nrf2.

**Figure 9:**
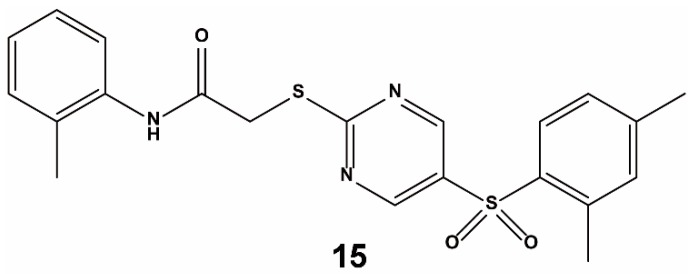
Structure of compound **15.**

Marcotte et al. combined a library of 1911 compounds from virtual screening with an Evotec Lead Discovery library containing 267,551 compounds for HTS [63]. The compounds (1911) were selected from three suppliers by docking to the Kelch domain of Keap1 (PDB ID: 1X2R) as performed with Glide. The combined chemical library was first screened via HTS using a homogeneous confocal fluorescence anisotropy assay. After primary screening, compounds lower than 79% binding activity at 50 µM were retested to confirm their inhibition. Then, a counter-screening assay was performed to confirm the selectivity of the hits in first-round screening. Finally, 18 top candidates were identified. Representative molecules from each cluster were chosen for screening against Nrf2–Keap1 in a biochemical assay. From this screening campaign, compound **15** (Figure 9) emerged as a lead compound against the Keap1–Nrf2 PPI. Compound **15** blocks the Nrf2 peptide binding site, inserts side-by-side into the central space of Keap1, and forms multiple hydrogen bonds and interacts with the π–π stack of several key residues, rather than through covalent adduct formation. The binding activity of compound **15** to the Keap1 protein was not very strong, however, and the EC_50_ value was a relatively high 118 μM.

### 3.2. Fragment-Based Approach

**Figure 10:**
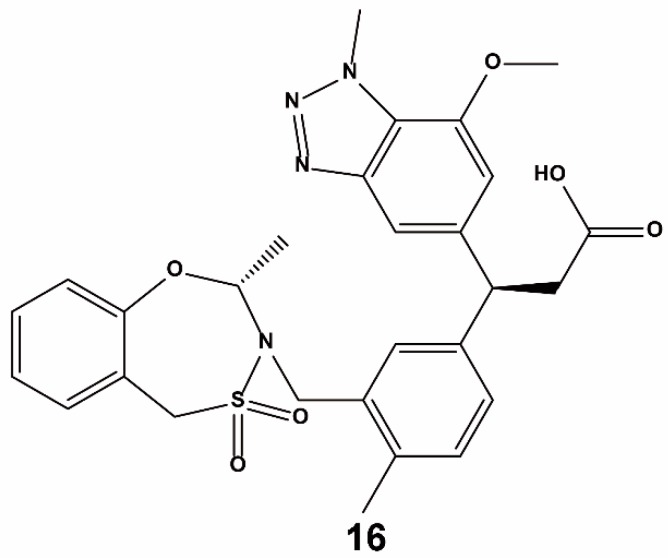
Structure of compound **16**.

Davis et al. reported the use of a fragment-based drug-design method for identifying Nrf2–Keap1 inhibitors [65]. To facilitate X-ray crystallography as the primary screen, a murine Kelch crystal structure was used which bears an unoccluded Nrf2 binding site. Screening of about 330 fragments via high-throughput soaking resulted in the observation of three different hotspots inside the Nrf2 binding site, near R483, Y525, and S602, respectively. Compound **16** (Figure 10), which was synthesized from the fragment hits, was identified as a strong and specific antagonist of the Keap1 Kelch–Nrf2 PPI. Compound **16** showed benefits in cellular and animal models of respiratory diseases induced by oxidative stress. In human bronchial epithelial cells, compound **16** increased Nrf2-regulated NQO1 expression and NQO1 enzymatic activity. However, these effects were inhibited when cells were co-treated with siRNA targeting Nrf2. A similar increase of Nrf2-regulated gene transcription was detected in the lungs of rats treated with compound **16**, demonstrating in vivo efficacy. Significantly, compound **16** also enhanced gene expression in bronchial epithelial cells from patients with chronic obstructive pulmonary disease (COPD), offering a connection to clinical disease states. Glutathione (GSH), a molecule that defends cells from oxidative stress, was recovered in a dosage-dependent fashion by compound **16** both in vitro and in vivo. Collectively, these results suggest that compound **16** stimulates the Nrf2 system leading to increase of target gene expression and promotion of subsequent downstream antioxidant and anti-inflammatory effects. Compound **16** is a promising lead compound for the treatment of defective Nrf2 pathway-activated diseases. Heightman et al. also identified compound **16** as a potent Nrf2–Keap1 inhibitor in vitro and *in cellulo* through a medicinal chemistry campaign based on combining a fragment-based method and optimization of ligand conformation [76].

### 3.3. Others

Schaap et al. reported a fluorescence resonance energy transfer (FRET)-based approach to screen Nrf2–Keap1 PPI inhibitors [77]. This assay measures the interaction between a yellow fluorescent protein (YFP)-bearing Keap1 Kelch binding domain with a cyan fluorescent protein (CFP)-bearing 16-mer peptide containing the Nrf2 ETGE region. Zhou et al. developed a screening strategy which combined FRET and a bimolecular fluorescence complementation (BiFC) assay [78]. This strategy can effectively screen for inhibitors of the Nrf2–Keap1 PPI that are potent in vitro and *in cellulo*. Hancock et al. reported a phage peptide display library using an FP assay and identified a peptide (Ac-DAETGEF-OH) Nrf2–Keap1 PPI inhibitor with an IC_50_ value of 0.73 μM [79].

## 4. Non-Screening Approaches for the Identification of Keap1–Nrf2 Interaction Inhibitors

**Figure 11:**
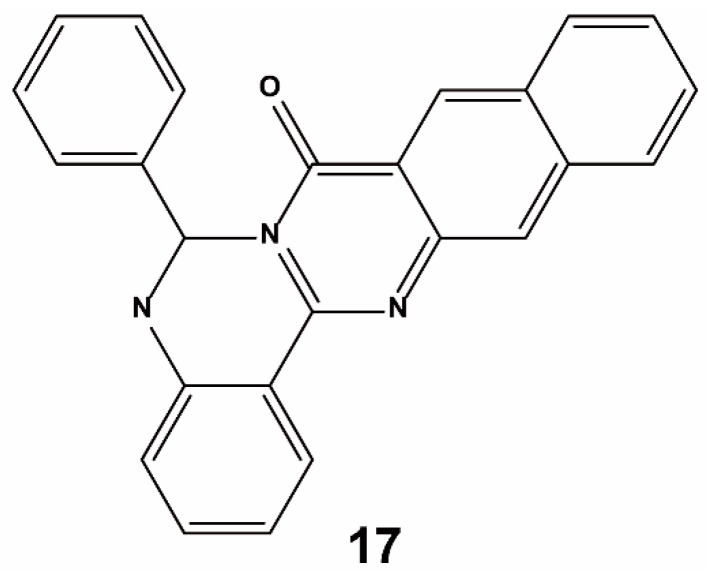
Structure of compound **17**.

Ghorab et al. performed a quantitative bioassay and a docking study to develop potent Keap1–Nrf2 PPI inhibitors. Molecular docking studies were performed using the Molecular Operating Environment (MOE) software version 10.2009 (Montreal, Quebec, Canada) against the X-ray structure of the Kelch domain of Keap1 (PDB ID: 4IQK) [80]. Protein targets were prepared for docking analysis by adding missing hydrogens and calculating partial charges. Self-docking calculations were performed with the target protein remaining rigid, while the ligand was free to rotate within the protein cavity. After performing multiple distinct docking simulations, the optimal conformation was selected based on the combination of the S-score data, the E-conformation (describing the absolute stereochemistry of double bonds in organic chemistry), and the appropriate fit to the relevant residues of the binding site. Finally, a series of quinazoline compounds with 1-phenyl-1,3,4-triazole scaffolds were reported by evaluating the ability of all compounds to induce the cytoprotective enzyme NQO1 using a quantitative bioassay. For assessing the capability of the synthesized molecules to target the Kelch domain of Keap1, an in silico experiment was conducted by docking against Keap1 (PDB ID: 4IQK). The main interactions observed between the native ligand and the protein target were arene–cation interactions with R415, an arene–arene linkage to Y525, and three hydrogen bonds with S602, S508 and S555. Upon docking of the synthesized compounds, they all showed an arene–cation binding interaction with Arg415 via their aromatic rings and one of the nitrogen atoms of the quinazoline ring. Compound **17** (Figure 11) emerged as the most potent inducer in this series, with activity in the nanomolar range. The compound has a unique structure with structurally rigid substituents and binds to critical residues within the binding location.

**Figure 12:**
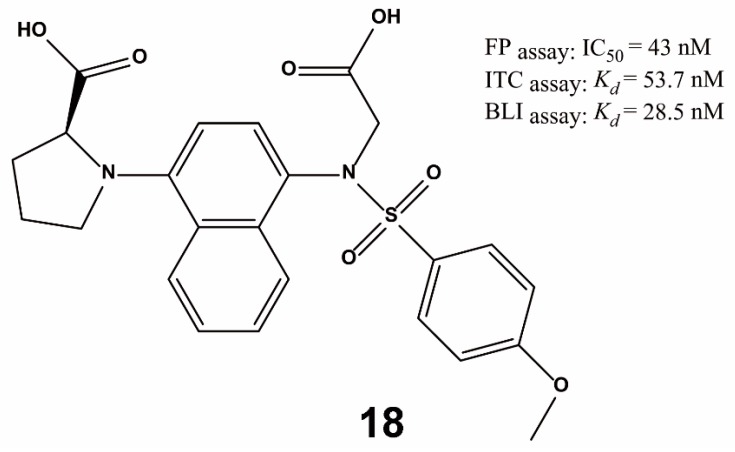
Structure of compound **18**. FP = fluorescence polarization; *K_d_* = equilibrium dissociation constant; ITC = isothermal titration calorimetry; BLI = biolayer interferometry.

Lu et al. designed and characterized a potent Keap1–Nrf2 inhibitor via preferentially substituting amino acids of reported inhibitors [81]. After performing a comprehensive structure–activity analysis, 37 compounds were designed and synthesized. The proline analogue **18** (Figure 12) was identified as a potent Keap1–Nrf2 PPI inhibitor with an IC_50_ of 43 nM via FP, and a *K_d_* value of 53.7 nM for Keap1 as measured by isothermal titration calorimetry (ITC). Compound **18** showed tight and prolonged Keap1 binding in vitro and *in cellulo* and could active Nrf2-regulated cytoprotective effects as well as reduced acetaminophen-induced liver injury in both cellular and animal systems. Besides offering a useful scaffold to further study the medicinal potential of Keap1–Nrf2 inhibition, this study also adds to the variety of chemical scaffolds capable of targeting the Keap1–Nrf2 interaction.

**Figure 13:**
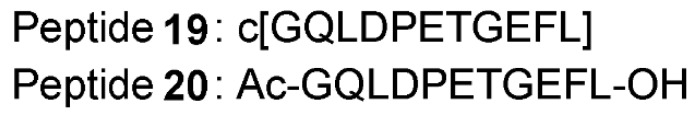
Sequences of compounds **19–20**.

Lu et al. also developed a cyclic peptide inhibitor of the Keap1–Nrf2 PPI using a head-to-tail cyclic strategy [82]. The cyclic peptide **19** (Figure 13) exhibited improved binding affinity to Keap1 than its corresponding linear peptide **20**, with *K_d_* values of 18.12 nM and 86.96 nM as measured by ITC, respectively. The affinities were corroborated by a biolayer interferometry (BLI) assay (*K_d_* value of compound **19** vs. **20** = 6.19 nM vs. 20.7 nM). In vitro Keap1–Nrf2 PPI inhibition was evaluated using the FP assay, and compound **19** exhibited an IC_50_ of 18.31 nM, making it more potent than compound **20** (IC_50_ = 63.15 nM). A cellular assay also indicated that compound **19** exhibited superior potency in activating Nrf2 and inducing antioxidant effects.

**Figure 14:**
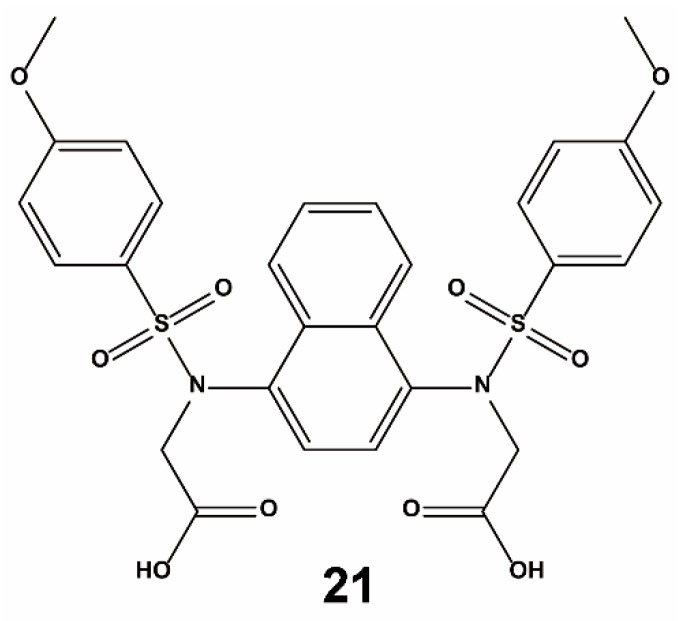
Structure of compound **21**.

Jiang et al. designed and identified compound **21** (Figure 14) as an effective antagonist of the Keap1–Nrf2 PPI based on structure-based design and molecular binding determinants analysis [83]. In the in vitro assay, compound **21** could directly bind to Keap1 and showed a *K*_d_ value of 9.91 nM. It also successfully disrupted the Nrf2–Keap1 interaction with an EC_50_ of 28.6 nM. In a cell-based ARE–luciferase reporter assay, compound **21** activated Nrf2 transcription in a dose-dependent manner, which was also supported by a qRT-PCR experiment.

## 5. Discussion

Extensive research on Nrf2–Keap1 signaling has been performed to elucidate the roles of this PPI in various diseases caused by oxidative stress, including cancer, Alzheimer’s disease (AD), chronic kidney disease, and diabetes. Targeting this PPI is thus emerging as a promising target for treating these diseases. To develop new and selective inhibitors of this PPI, efficient screening strategies are urgently needed for their identification [29,84,85]. Each screening strategy has different benefits and drawbacks which should be considered before their use (Table 2).

Progress in structural biology has uncovered new opportunities for developing small molecule antagonists of the Keap1–Nrf2 PPI using SBVS approaches. The advantage of SBVS over LBVS is that it is possible to find novel inhibitors with new structures and unique mechanisms of action, because SBVS is based on the nature of the target itself without considering the structures of known ligand molecules, which makes it possible to conduct SBVS against a completely new drug target. However, SVBS is more computationally intensive than LBVS techniques [86], and it is difficult to obtain genuine Keap1–Nrf2 PPI inhibitors due to off-target side effects caused by ignoring the ligands of the target. Therefore, the combination of SVBS and LBVS may be more effective in screening very large compound databases in an efficient manner. Meanwhile, SPR-, ITC- and BLI-based screening approaches are label-free and have low false positive rates, but their drawbacks are their high cost, low-throughout, and high technical and equipment requirements, which restricts their widespread use for screening [87]. As for fluorescence-based approaches, such as the BIFC-based assay, FP assay, FCS-based assay, and TR-FRET assay, they are generally simple and cheap to perform, but can be susceptible to interference by compounds with autofluorescence or fluorescence quenching ability. Non-screening approaches are often based on reported lead compounds to develop analogues with improved potency and selectivity. However, these methods are unable to discover new scaffolds and increase chemical diversity.

Although many reported chemical leads against the Keap1–Nrf2 PPI have been reported in the literature with good inhibitory potencies, electrophilic Nrf2 activators may lack selectivity and thus lead to off-target side effects. Thus, the continual development of new classes of selective Keap1–Nrf2 PPI inhibitors are required. In particular, their efficacies and selectivities in vivo need to be more rigorously verified. Up to now, only a few disease models related to the Keap1–Nrf2 PPI have been evaluated in lab and clinical research, such as mouse renal inflammation [88,89], Huntington’s disease [90], drug-induced liver injury [84,91], and many cancer models [92,93,94,95,96,97,98]. Other disease models, such as diabetes [99], neurodegenerative disease [5,46,100,101,102], and cardiovascular diseases [50] may be considered in future studies.

As we have highlighted in this review, recent efforts to target the Keap1–Nrf2 PPI have been promising. We encourage researchers to increase their hit rate by using effective drug screening methods. Meanwhile, it is also important to open up new sources of chemical scaffolds to broaden the diversity of chemotypes as Keap1–Nrf2 PPI inhibitors, including diversity-oriented libraries or natural product-like libraries. We hope that the first Nrf2–Keap1 PPI inhibitor will soon enter the clinic for the treatment of human diseases.

## Figures and Tables

**Table 1:** Comparison of different strategies used in the screening of Nrf2–Keap1 PPI inhibitors.

Method	Library Size	Screening Software	Ref
High-throughput screening (HTS)	2000 compounds	-	[60]
	1633 drugs	-	[61]
Virtual screening (VS)	251,774 compounds	Ligandfit	[10]
	~178,000 compounds	Autodock 4.2 and DOCK 6.6	[62]
HTS + VS	(267,551 + 1911 compounds)	Glide 5.5	[63]
	300,000 compounds	Schrodinger’s Glide	[64]
Fragment-based approach	330 molecular fragments	-	[65]

**Table 2:** Comparison of different approaches for developing Keap1-Nrf2 PPI inhibitors.

Methods	Benefits	Drawbacks
VS	Low cost; high-throughput	High false positive rate; only applicable for primary screening
SPR ^a^	Label-free; low false positive rate	Low-throughput; high technical and equipment requirements; high cost
ITC		
BLI		
BIFC-based assay [79] ^b^	High-throughput; label-free; low false positive rate	Low sensitivity
ARE gene promoter assay [61] Phage peptide display [79] ^c^		Low sensitivity
FP-based assay [79]	High-throughput, easy to operate; high sensitivity	Labeling requirement; interference by compounds with autofluorescence or fluorescence quenching ability
FCS-based assay [61] ^d^ TR-FRET-based assay [77,84] ^e^		

Notes: a. SPR = surface plasmon resonance; b. BIFC = Bimolecular fluorescence complementation; c. ARE = antioxidant response element; d. FCS = Fluorescence correlation spectroscopy; e. TR-FRET = Time-resolved fluorescence energy transfer.
